# Functional analysis of fibroblasts and macrophages in head and neck paragangliomas

**DOI:** 10.3389/fendo.2024.1397839

**Published:** 2024-11-15

**Authors:** Paramita Baruah, Jennifer L. Marshall, Meriam Nefla, Valentina Pucino, Holly Adams, Jason D. Turner, Sebastian Gilbert, Emily Powell, Georgiana Neag, Peter Monksfield, Richard M. Irving, Adam P. Croft, Ingrid E. Dumitriu, Christopher D. Buckley

**Affiliations:** ^1^ Department of Ear Nose and Throat (ENT), University of Leicester National Health Service (NHS) Trust, Birmingham, United Kingdom; ^2^ Department of Ear Nose and Throat (ENT), Queen Elizabeth Hospital Birmingham, Birmingham, United Kingdom; ^3^ Institute of Inflammation and Ageing, University of Birmingham, Birmingham, United Kingdom; ^4^ Birmingham Tissue Analytics, University of Birmingham, Birmingham, United Kingdom; ^5^ Cardiovascular Research Institute, University of Birmingham, Birmingham, United Kingdom

**Keywords:** fibroblasts, macrophages, head and neck paraganglioma, CD90, CD163, MCT1, MCT4

## Abstract

**Background and aim:**

Head and neck paragangliomas (HNPGN) are tumours that carry significant morbidity The role of the stroma in the pathogenesis of HNPGN is not completely understood. This study explores the profile of fibroblasts and macrophages in HNPGN.

**Methods:**

Ten patients undergoing HNPGN surgery were recruited. CD68 and CD163 immunohistochemistry was performed for macrophage analysis; CD90 and podoplanin (PDPN) expression was examined to identify fibroblasts. RT-qPCR was performed on HNPGN tissue for macrophage- and fibroblast-associated molecules. Fibroblast cultures were established from HNPGN were analysed by RT-qPCR and flowcytometry. Confocal microscopy for MCT1 and MCT4 was performed in HNPGN.

**Results:**

CD68 and CD163 expressing macrophages were noted in HNPGN. CD90 and PDPN expressing cells were present in HNPGN. RT-qPCR analysis showed expression of phenotypic and functional macrophage- and fibroblast-associated molecules in HNPGN. RT-qPCR analysis of fibroblasts cultured from HNPGN confirmed the expression of several molecules including PDPN at comparable levels to healthy tissue fibroblasts. Expression of FAP, MCT-1, insulin receptor (CD220) and insulin growth factor receptor-2 (CD222) was noted on HNPGN derived fibroblasts on flowcytometry. MCT1 and MCT4 were expressed in HNPGN tumour cells and stromal macrophages *in-situ*.

**Conclusion:**

Fibroblasts and macrophages are present in the HNPGN tumour microenvironment, and several macrophage and fibroblast functional markers are expressed in HNPGN. Macrophages in HNPGN tissue express metabolic markers MCT1 and MCT4. Further analysis of the fibroblast and macrophage function in HNPGN will improve our understanding of their potential roles in tumour pathogenesis.

## Introduction

Head and neck paragangliomas (HNPGNs) are neoplasms of the autonomic system arising from neural crest derived cells of the parasympathetic paraganglia of the skull base and neck (1). Also called glomus tumours, HNPGNs can arise in relation to the jugular vein (glomus tympanicum and glomus jugulare), cranial nerves (e.g. vagal paraganglioma) and the carotid artery (carotid body tumour). HNPGNs are usually slow growing tumours but their location at the base of the skull and propensity for intracranial expansion cause considerable morbidity (stroke, cranial nerve palsies with speech and swallowing impairment, facial palsy, hearing loss), sometimes with fatal outcomes. Growing HNPGNs are traditionally managed via surgery and/or radiotherapy, which carry significant morbidity and mortality due to proximity to the great vessels of the neck and several cranial nerves. It is therefore imperative to develop newer treatments to improve outcomes in patients with HNPGNs. In addition to the challenges described above in treating HNPGNs, their clinical behaviour is difficult to assess and predict. The benign versus malignant nature of HNPGNs remains a clinical conundrum as it cannot be determined based solely on histology or current imaging methods. Over 15% of HNPGNs are malignant but are discovered late in the course of the disease when patient develops metastases. Further diagnostic challenges arise when a patient presents with multiple paragangliomas simultaneously and it becomes difficult to determine if these are individual primary tumours or metastatic foci. The presence of regional metastases is associated with less than 60% survival rates and distant metastases further worsen the prognosis. The etiopathogenesis of HNPGNs is not completely understood. They can be sporadic but up to 30-50% of HNPGNs are hereditary and associate with germline mutations in various genes. Of note, about 50% of these mutations involve the succinate dehydrogenase (SDH) gene, which codes an enzyme integral to the Krebs cycle (1). Very little is known of the cellular and molecular mechanisms, and in particular the role of the stromal microenvironment in regulating HNPGN pathogenesis. A better understanding of the biology of the HNPGN tumour microenvironment is likely to reveal markers of growth and malignant behaviour and thereby enable early detection, targeted therapies and prevention of recurrences and metastases.

Tumour stroma has been shown to play a profound role in regulating tumour progression by supporting angiogenesis, tumour cell proliferation, invasion, metastasis and mechanisms of resistance to treatment (2). The role of the stromal microenvironment in HNPGN pathogenesis and, specifically, their functional profile has not been addressed so far. Tissue resident fibroblasts and macrophages are important components of tumour stroma. We have previously shown that VEGF, a pro-angiogenic factor, localises mainly in the stroma of HPV-positive head and neck cancers (3) and that HPV-positive cancer head and neck cancer cells can up-regulate programmed death ligands PD-L1 and PD-L2 on fibroblasts (4). We have also shown that fibroblasts derived from vestibular schwannomas exhibit a pro-tumorigenic profile (5). Tissue resident macrophages are known to undergo metabolic reprogramming in an inflammatory environment via the succinate dehydrogenase (SDH) pathway (6). This is of particular relevance in hereditary HNPGNs that display SDH mutations as alterations in cellular metabolism could drive HNPGN initiation and progression. There is evidence that cancer associated fibroblasts and macrophages undergo metabolic reprogramming, which supports tumour progression (7). Stromal cells may thus have hitherto unknown roles in the progression of HNPGN. In this study we show expression of fibroblast markers CD90 and PDPN in HNPGN tissue *in-situ* as well as presence of CD163 expressing macrophages. We isolate fibroblasts from HNPGN and confirmed the expression of CD90 *in vitro*. We further identified the expression of monocarboxylate transporters MCT1 in HNPGN derived fibroblasts by flowcytometry and the expression of MCT1 and MCT4 in tumour cells and macrophages *in situ* in HNPGN tissue. Further dissection of the phenotypic and functional profile of fibroblasts and macrophages in HNPGN will help decipher the tumour microenvironment in HNPGN with the aim of uncovering new risk stratification and therapeutic targets.

## Materials and methods

### Patient demographics

Ethical approval for the study was obtained from the University of Birmingham research ethics committee (Human Biomaterials Resource Centre HBRC 17-295) and the tissue samples were released via HBRC. Patients were recruited into the study following informed consent. HNPGN tissue was collected from ten patients undergoing tumour excision. Healthy tissue from the ear (mastoid mucosa and ear canal skin) was collected from 6 patients to culture fibroblasts. The diagnosis of HNPGN in the patients was made on clinical grounds and confirmed by histopathology in the Department of Pathology, University of Birmingham NHS Trust. The age range of the patients at surgery was 26-72 years with a mean of 43.2 years; 3 of the tissue samples originated from male and 7 from female patients. Clinical data obtained included patient age at operation, gender, SDH mutation status staging of the disease and clinical outcomes and is summarized in **Supplementary Table S1**.

### Human tissue processing and histology

Tumour tissue samples were frozen in Tissue-Tek OCT medium or formalin fixed and paraffin embedded (FFPE). For immunohistochemistry, antigen retrieval was performed at pH9. Sections were stained using polyclonal sheep anti-human CD90 (AF2067, R&D) and rat anti-human podoplanin (PDPN) (Clone NZ-1.3, eBioscience), Mouse anti-CD163 (clone 10D6, Leica) or Mouse anti-CD68 (Leica, clone 514H12) for one hour at room temperature. For the DABs and Red staining, Leica Bond Polymer Refine Detection systems were used. Nuclei were counterstained with Hematoxylin QS (Vector Laboratories). Images were acquired using the Zeiss Axio Scan and analysed with QuPath software (v0.4.3). Visiopharm quantitative analysis was performed to threshold the DAB and FsstRed on images, after thresholding for Haematoxylin. Marker positive area (in µm^2^) and nuclei area (in µm^2^) were measured, and the ratio calculated for each sample studied. Co-relation analyses were performed based on tumour stage/gender and mutation status.

For the MCT1/MCT4 and CD90/CD68 staining, after the antigen retrieval step (45 minutes) and block of non-specific binding (1 hour), paraffin-embedded tissue sections were incubated at 4°C for 1 hour with the following antibodies: rabbit polyclonal anti-MCT1(1:300, Bethy), mouse monoclonal anti-MCT4 (1:100, Santa Cruz), polyclonal sheep anti-CD90 (1:100, R&D), biotin anti-CD68 (1:100, Novus). Streptavidin Alexa Fluor™ 594, donkey anti-sheep IgG Alexa Fluor™ 546, donkey anti-Rabbit IgG AlexaFluor™ 488, donkey anti-Mouse IgG Alexa Fluor™ 647 were used as secondary antibodies (1:300). Hoechst was used for staining nuclei. Slides were mounted with Prolong Gold Antifade reagent (Invitrogen). Images were acquired on a confocal microscope (Zeiss LSM 780) and analysed using Zen software.

### Human fibroblast cell culture

Primary human fibroblasts were isolated as described (8, 9) and cultured in RPMI (Sigma-Aldrich) with heat inactivated 10% foetal calf serum (FCS; Labtech International, Sussex, UK), L-glutamine, Sodium Orthopyruvate (Sigma Aldrich), antibiotics (penicillin and streptomycin), and MEM non-essential amino acids (Sigma Aldrich). Passage 1 fibroblast lines at were used for the RT-qPCR experiments. Flowcytometry was performed on fibroblasts at passages 2-5.

### Quantitative reverse transcription PCR

RT-qPCR was carried out using customised macrophage and fibroblast panels (Applied Biosystems). RNA was isolated from frozen tissue or frozen fibroblasts at passage 1 using the RNAeasy RNA isolation kit (Qiagen) according to the manufacturer’s instructions. cDNA synthesis was performed on all samples (500 ng of RNA was transcribed) using SensiFAST cDNA Synthesis Kit (Bioline) on a Mastercycler (Eppendorf) thermal cycler PCR machine. Reverse transcription with quantitative PCR (RT−qPCR) was performed using a Taqman Gene Expression array and Taqman universal Mastermix on the ABI 7900 real-time PCR detection system (both Applied Biosystems) and using the TaqMan Array Microfluidic Card. Expression levels were normalized to an internal housekeeping gene (GAPDH) and a relative amount of expression for genes of interest was calculated form the delta CT to the housekeeping gene (2^-^ΔCT) The primers used in the arrays (Fibroblast panel) were: EGF-Hs01099999_m1, FGF2-Hs00266645_m1, TP53BP2-Hs00610488_m1, HTR2A-Hs00167241_m1, TNS3-Hs00224228_m1, EPAS1-Hs01026149_m1, HIF1A-Hs00153153_m1, PADI4-Hs00202612_m1, MYC-Hs00153408_m1, RAF1-Hs00234119_m1, SYVN1-Hs00381211_m1, INHBA-Hs00170103_m1, CXCL12-Hs00171022_m1, GAPDH-Hs99999905_m1, B2M-Hs00187842_m1, VCAM1-Hs01003372_m1, CD248-Hs00535586_s1, PDPN-Hs00366766_m1, LGALS1-Hs00355202_m1, LGALS9-Hs00371321_m1, LGALS3-Hs00173587_m1, LGALS12-Hs00263821_m1, CXCL16-Hs00222859_m1, 18S-Hs99999901_s1, MMP1-Hs00899658_m1, MMP2-Hs01548727_m1, MMP3-Hs00968308_m1, MMP9-Hs00234579_m1, MMP13-Hs00233992_m1, CTSL1-Hs00377632_m1, CTSB-Hs00947433_m1, SUMO1-Hs02339312_g1, S100A4-Hs00243201_m1, DKK1-Hs00183740_m1, NAMPT-Hs00237184_m1, MMP14-Hs00237119_m1, CTSK-Hs00166156_m1, IGF2-Hs00171254_m1, ACTA2-Hs00909449_m1, TLR2-Hs01014511_m1, TLR3-Hs00152933_m1, TLR4-Hs00152939_m1, TNFSF11-Hs00243522_m1, LGALS3BP-Hs00174774_m1, SLIT3-Hs00171524_m1, TRERF1-Hs00363301_m1, SHOX-Hs00230846_m1, PIAS1-Hs00184008_m1, EGF-Hs01099999_m1. For the macrophage RT-qPCR panel the primers used in the arrays were for the following genes of interest: STAT-1, ALOX15, INHBA, CCL2, CCL5, IL8, CxCL10, CD64, SPl1, CD32, IL6, IL10, TNF, IL1b, RANK, MRC1, PTPRC, EpCAM, ACP5, CTSK, CD68, RPL13A, MERTK, HLADRA, CD163, CD14, FN1, Thy1(CD90), PDPN, CD80, CD16b, MCT4, MCT3, Cav1, HIF1a, CSF1R, CSF2RA and IDO.

### Flow-cytometry

For detection of CD90 and PDPN, cultured fibroblasts were detached by incubation with Trypsin/EDTA (Sigma-Aldrich) at 37°C, followed by washes in culture medium. Cells were washed several times in PBS with 2% FCS and stained with the following antibodies: anti-CD90 (PerCP-Cy5.5 conjugated (eBio5E10), eBioscience), PDPN (PE conjugated (NZ-1.3), eBioscience) and anti-FAP (Fibroblast Activation Protein) (sheep anti-human, R&D). Staining was also performed with MCT1 (Rabbit polyclonal, Bethyl), MCT4 (D-1, Santa Cruz), PE anti-human CD220 (clone REA260, Miltenyi Biotec) and PE anti-human CD222 (clone REA187, Miltenyi Biotec). Fixable Viability Stain 575V (BD Biosciences) was used to exclude dead cells from the analysis. Samples were acquired on a Beckman Coulter CytoFLEX flow cytometer and data analysis was performed using FlowJo software version 10.

### Statistical analysis

Statistical analysis was performed using GraphPad Prism software version 10.0.3. RT-qPCR data on fibroblasts derived from HNPGN and normal tissue were compared using two tailed Mann-Whitney test. Correlation analysis of expression level with tumour stage were performed with the Spearman’s test. Analysis of expression level with gender or mutation status was performed with Mann-Whitney test. Probability values (p) of less than 0.05 were considered statistically significant.

## Results

### Fibroblasts and macrophages in HNPGN tissue on immunohistochemistry

**Figure 1:**
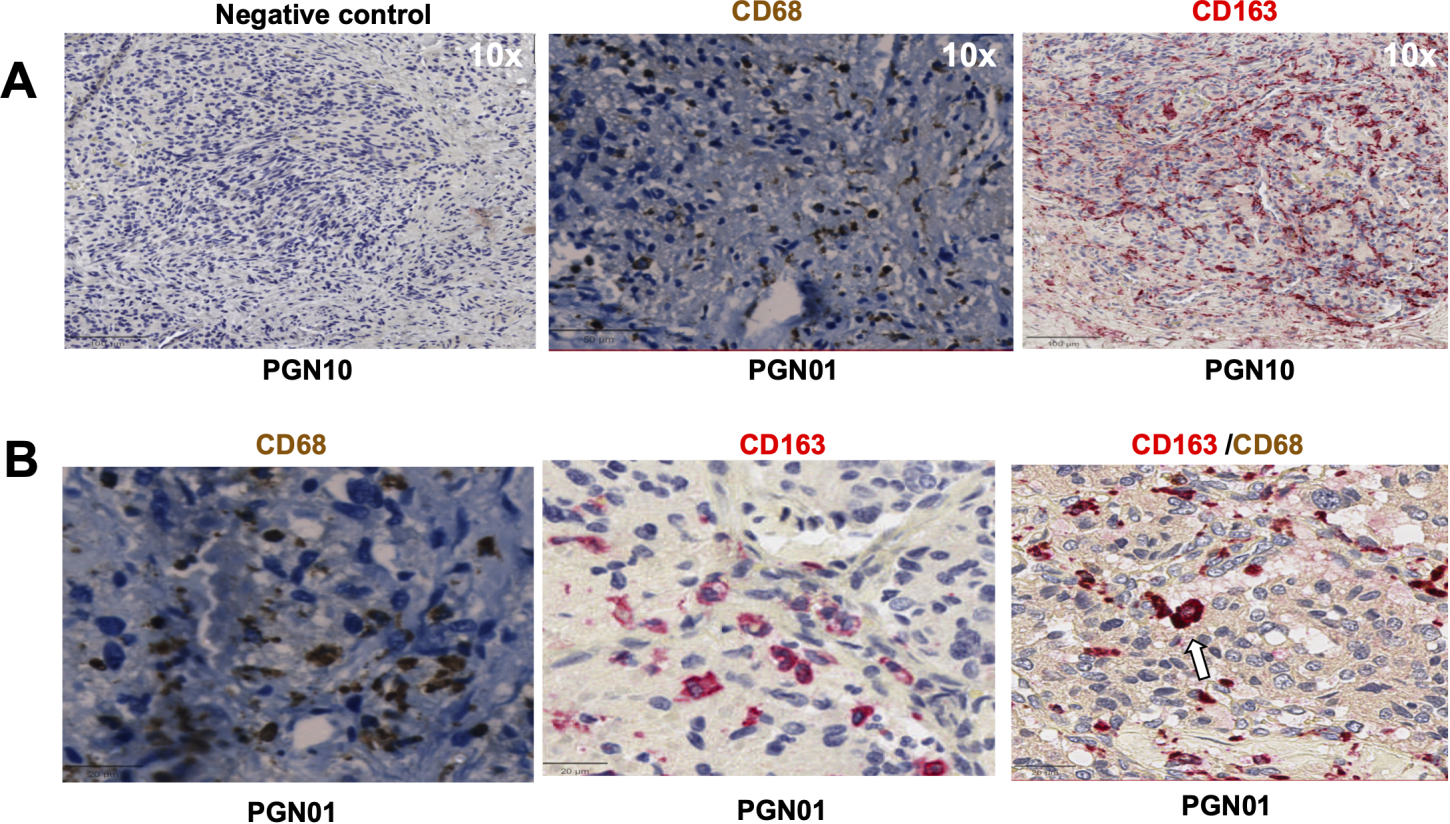
Expression of CD68, CD163 in head and neck paraganglioma (HNPGN) tissue on immunohistochemistry. Immunohistochemistry images of HNPGN tissue (representative of n = 10 HNPGN patient samples). **(A)** Control antibody staining (left panel, negative control, patient sample PGN10), CD68 staining (middle panel, patient sample PGN01) and CD163 staining (right panel, patient sample PGN10). CD68 is in dark brown, CD163 is in red and nuclei are blue. **(B)** Double staining with CD68 (dark brown) and CD163 (pink). CD68/CD163 double co-expressing macrophages (arrow). Images are at a magnification of 20x.

**Figure 2:**
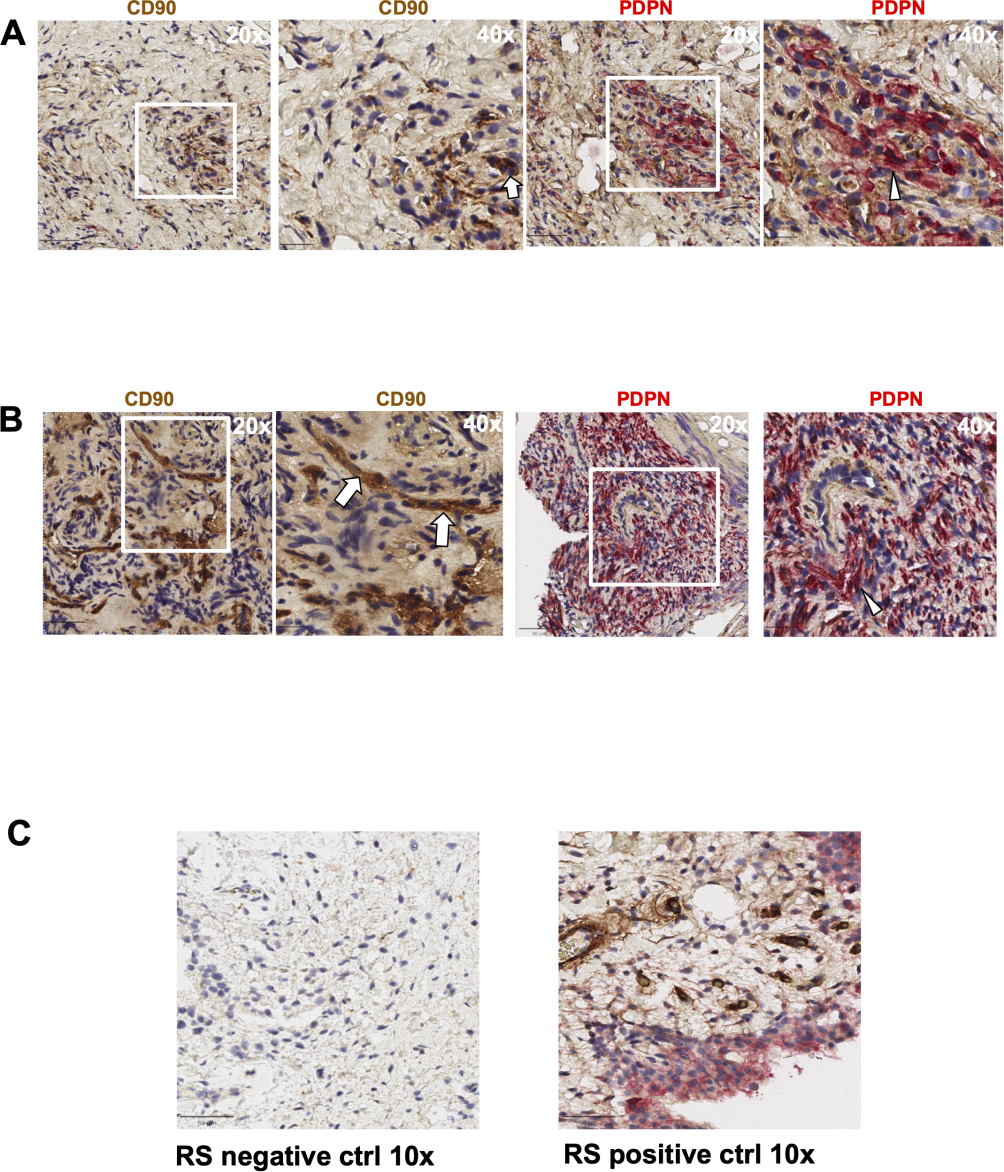
Expression of PDPN and CD90 in head and neck paraganglioma (HNPGN) tissue on immunohistochemistry. Immunohistochemistry images of HNPGN tissue. CD90 is in brown and podoplanin (PDPN) is in pink, nuclei are blue. **(A)** PDPN and CD90 expression in HNPGN sample from patient PGN010. Left panel is at 20x magnification while the right panel show 40x magnification of the tissue area in the white box inset. **(B)** PDPN and CD90 staining as above in HNPGN tissue from patient PGN09. Arrows point to CD90 stained cells and arrowheads to the PDPN stained cells **(C)** Control staining (left panel) and positive staining (right panel) with CD90 (brown) and PDPN (pink) is displayed in tissue from synovium in rheumatoid arthritis (RA).

We first evaluated the presence of macrophages and fibroblasts in HNPGN tissue from ten patients using immunohistochemistry(IHC). CD68 and CD163 staining was performed as they are canonical macrophage markers- CD68 is a pan macrophage marker and CD163 is a M2 macrophage marker (10). CD68 expression was noted in HNPGN indicating macrophage infiltration (**Figure 1A**, middle panel). Furthermore, CD163 expression was noted in all ten macrophage samples (**Figure 1**; **Supplementary Figure S1**). Double staining with CD68 and CD163 on immunohistochemistry was performed and co-localisation noted indicating M2 polarisation of macrophages in HNPGN (**Figure 1B**). We next examined the expression of CD90 and podoplanin (PDPN), both of which are expressed by fibroblasts (11). CD90 expression was noted in elongated nucleated cells in stromal areas of the HNPGN in keeping with fibroblasts. Similarly, PDPN expression was noted in cells in the stromal region (**Figure 2**). The two markers were however not found to co-localise. A quantitative analysis of expression of CD163, CD90 and PDPN was performed and was analysed for correlation with stage, gender and mutational status. No significant correlation was found with the tumour stage and expression of CD163, CD90 and PDPN in this cohort (CD90, r=0.04, p=0.9; PDPN, r = -0.4, p=0.19; CD163, r=0.27, p=0.43). Further comparison of the expression of these markers versus gender and mutation status did not show significant differences (**Supplementary Figure S4**).

### Fibroblast and macrophage related markers in HNPGN tissue on RT-qPCR

Having confirmed the presence of macrophages and fibroblasts in HNPGN tissue we performed RT-qPCR on fresh frozen HNPGN tissue to study a panel of fibroblast-associated and macrophage-associated markers (details in Materials and Methods). PDPN (**Supplementary Figure S5**, upper panel) and CD90 (Thy 1) (**Supplementary Figure S5**, lower panel) were also found to be expressed in HNPGN tissue on RT-qPCR. Similarly, macrophage markers CD68 and CD163 were expressed in HNPGN tissue on RT-qPCR (**Supplementary Figure S5**, lower panel). In addition, several molecules belonging to the Galectin family (LGALS1, LGALS3, LGALS9, LGALS3BP), Toll-like receptors (TLR2/TLR4) and matrix metalloproteinases (MMP2/MMP9/MMP14) were expressed in HNPGN tissue (**Supplementary Figure S5**, upper panel). HNPGN associates with SDH mutations which could result in metabolic changes within the HNPGN microenvironment. In keeping with this, markers of cellular metabolism such as MCT4 and HIF-1α were found to be expressed in HNPGN tissue on RT-qPCR (**Supplementary Figure S5**, lower panel).

### Fibroblast related markers in HNPGN derived fibroblasts on RT-qPCR

We next compared fibroblasts derived from HNPGN to healthy tissue (mastoid mucosa and/or ear canal skin) fibroblasts to verify the expression of fibroblast- associated molecules that were identified in the RT-qPCR in HNPGN tissue. For this we first cultured fibroblasts from HNPGN tissue (described in Materials and Methods). Fibroblast cultures were successfully established from all the ten HNPGN samples (**Supplementary Figure S6A** left panel). Fibroblast cultures were also successfully established from mastoid mucosa and/or ear canal skin (healthy tissue) from six patients (**Supplementary Figure S6A** right panel). RT-qPCR analysis was undertaken on the cultured HNPGN fibroblasts and healthy tissue fibroblasts with the panel of fibroblast markers performed previously on the HNPGN tissue. Results from paired samples (HNPGN and normal tissue fibroblasts from the same patient) of tumour and healthy tissue fibroblasts are presented (**Supplementary Figure S6A**). Levels of LGALS1 and MMP14 showed a higher expression trend in HNPGN fibroblasts but did not reach significance. Other molecules such as LGALS9, LGALS3BP, MMP2 etc were similar in the two groups.

### Fibroblast associated markers in HNPGN derived fibroblasts on flow-cytometry

**Figure 3:**
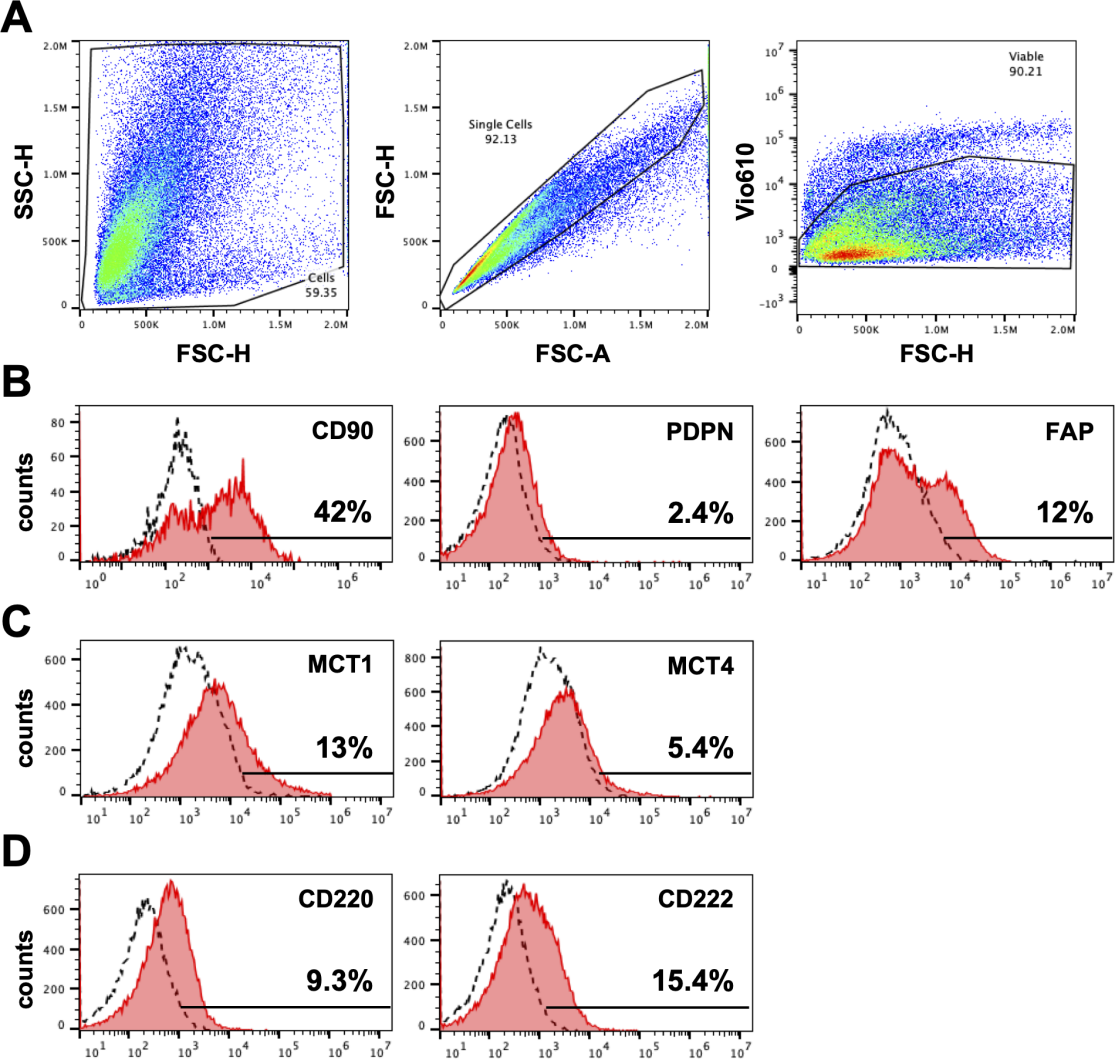
Expression of markers on fibroblasts derived from head and neck paraganglioma (HNPGN). Expression on fibroblast associated markers were examined by fibroblasts cultured from HNPGN using flow cytometry. **(A)** The sequential gating strategy is illustrated based on forward scatter and side scatter (left panel), exclusion of doublets (middle panel) and exclusion of dead cells (Fixable viability stain 575V negative population, right panel). **(B)** Illustrative histogram plots depicting the expression of CD90, PDPN and FAP (pink histograms). Dashed histograms show staining with isotype control antibodies. **(C)** Illustrative histogram plots showing the expression of MCT1 and MCT4 (pink histograms). **(D)** Illustrative histogram plots showing expression of CD220 AND CD222 (pink histograms). Dashed histograms depict staining with isotype control antibodies. Representative of fibroblasts from three different HNPGN samples.

We next evaluated expression of fibroblast markers on the cultured cells using flowcytometry. In keeping with our immunohistochemistry findings, cultured fibroblasts from HNPGN were found to express CD90 (fibroblast marker) on flowcytometry (**Figure 3**) Very low PDPN expression was noted in the cultured fibroblasts (**Figure 3**). FAP (Fibroblast activation protein) expression was observed in a fraction of the cultured fibroblasts. As HNPGN is frequently associated with SDH mutations and we found MCT4 expression in HNPGN tissue on RTPCR, we also examined the cultured fibroblasts for expression of members of the metabolic pathway. Approximately 13% of the cultured fibroblast population expressed metabolic marker monocarboxylate transporter-1 (MCT1) while monocarboxylate transporter-4 (MCT4) expression was lower. Similarly, about 10% of cultured fibroblasts expressed insulin receptor (CD220) and about 15% expressed insulin-like growth factor 2 receptor (CD222) (**Figure 3**).

### Expression of metabolic markers MCT1 and MCT4 in HNPGN tissue

**Figure 4:**
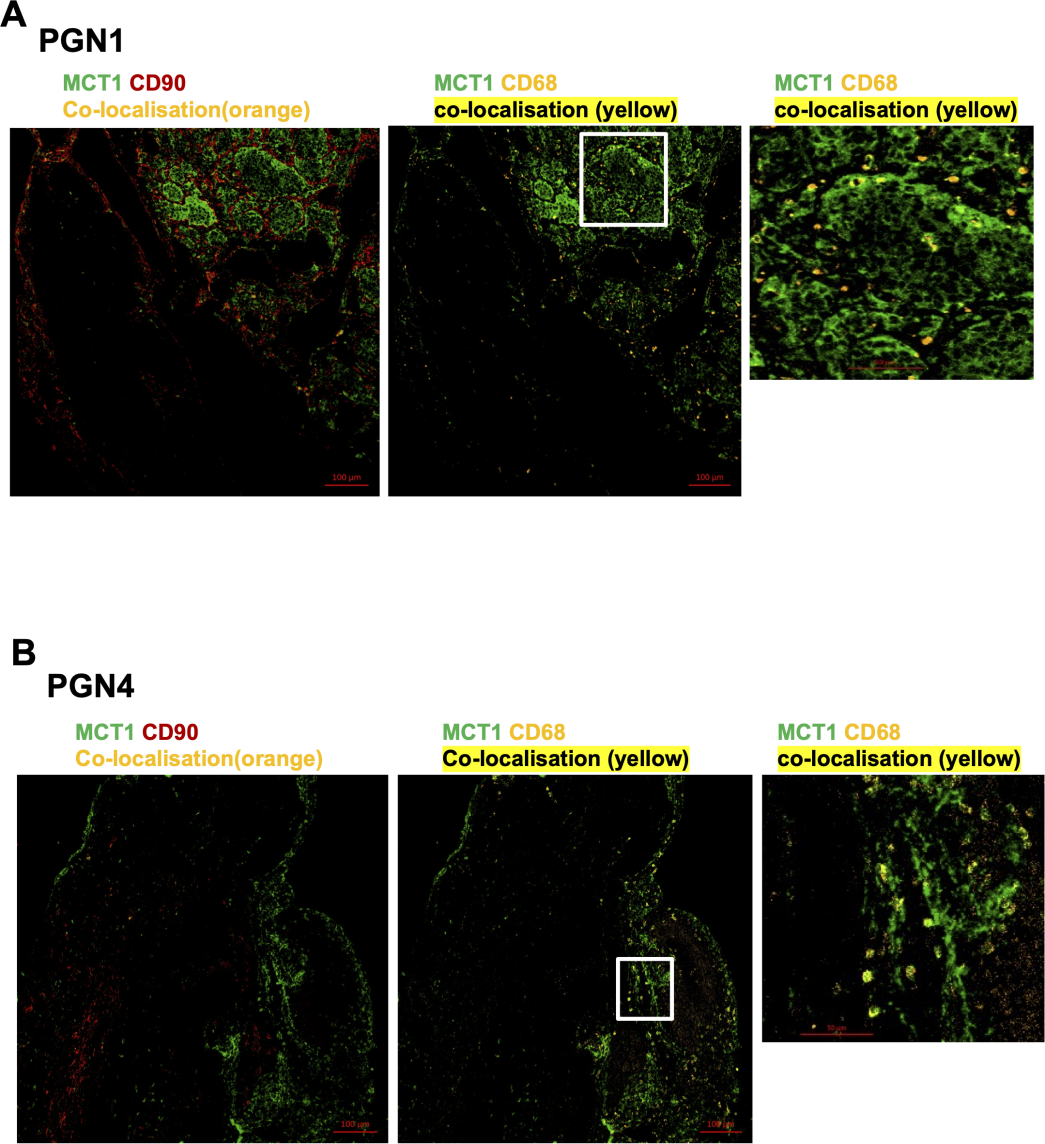
Expression of Monocarboxylate transporter 1 (MCT1) in head and neck paraganglioma (HNPGN). HNPGN tissue was stained with fluorochrome-labelled antibodies to assess lactate transporter MCT expression. **(A)** Representative immunofluorescence staining of MCT1 (green) expression in HNPGN tissue from patient PGN01 (no SDH mutation). Fibroblasts are in red (CD90) and macrophages in orange (CD68). MCT1 co-localisation with CD68 is in yellow. Right side small panel is a magnified image of the tissue area in the white box inset showing CD68 and MCT1 co-expressing macrophages in yellow. (n=3 HNPGN tissue). **(B)** Representative immunofluorescence staining of MCT1 (green) expression in HNPGN tissue from patient PGN04 (with SDH mutation). Staining as in **(A)**. (n=3 HNPGN tissue).

**Figure 5:**
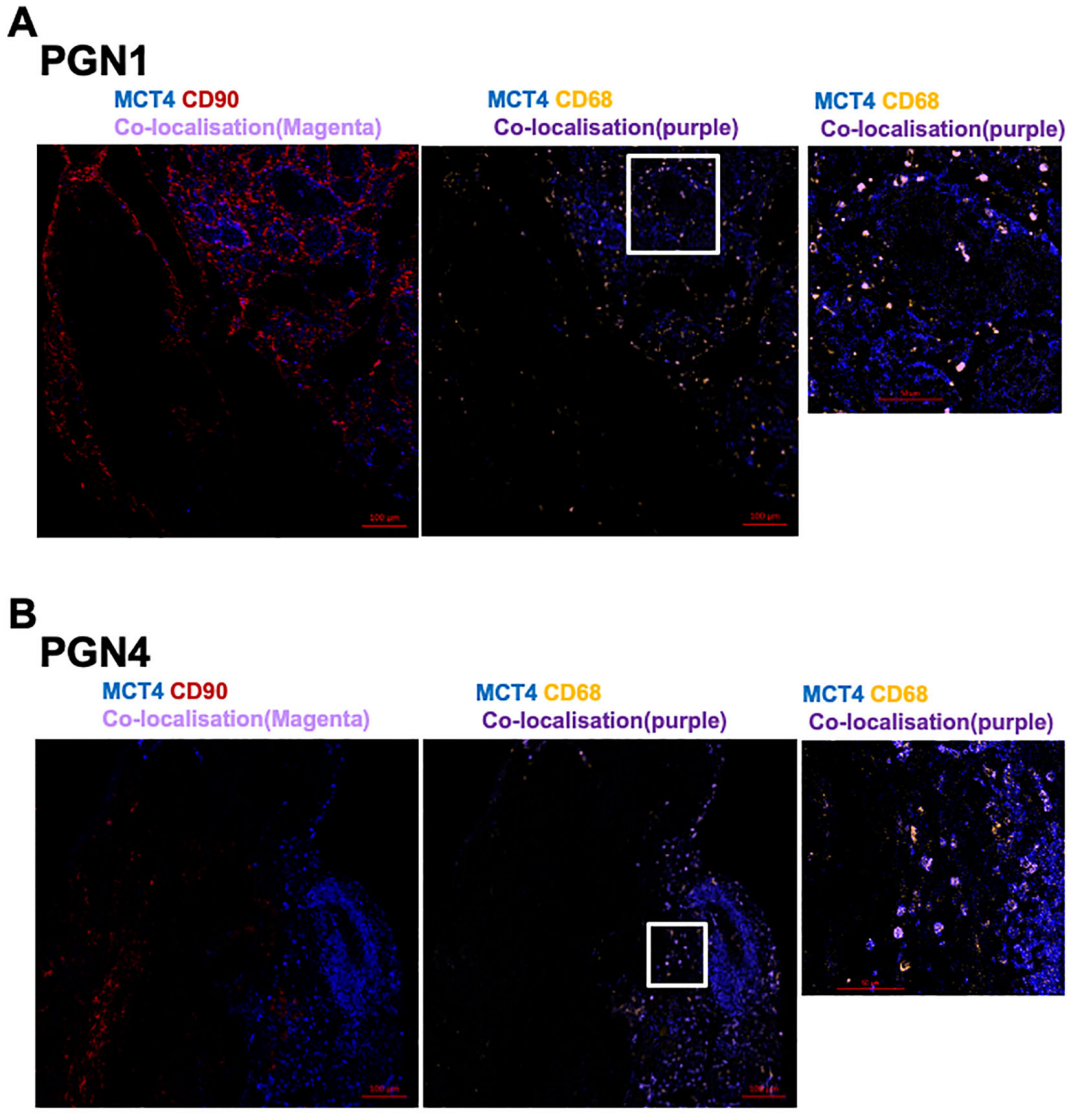
Expression of Monocarboxylate transporter 4 (MCT4) in head and neck paraganglioma (HNPGN). HNPGN tissue was stained with fluorochrome-labelled antibodies to assess lactate transporter MCT4 expression. **(A)** Representative immunofluorescence staining of MCT4 (blue) expression in HNPGN tissue from patient PGN01 (no SDH mutation). Fibroblasts are in red (CD90) and macrophages in orange (CD68). MCT4 co-localisation with CD68 is in purple. Right side small panel is magnified image of the tissue area in the white box inset showing CD68 and MCT4 co-expressing macrophages (in purple). (n=3 HNPGN tissue). **(B)** Representative immunofluorescence staining of MCT4 (blue) expression in HNPGN tissue from patient PGN04 (with SDH mutation). Staining as in **(A)** (n=3 HNPGN tissue).

MCT1 and MCT4 are plasma membrane transporters involved in the transport of lactate and pyruvate (12). We found expression of MCT4 in HNPGN tissue on RT-qPCR (**Supplementary Figure S2**). In addition, we found MCT1 expression in fibroblasts derived from HNPGN on flowcytometry (**Figure 3**). We therefore examined the presence of MCT1 and MCT4 in HNPGN tissue using immunofluorescence. Three samples from HNPGN patients with germline SDH mutations and 3 samples from HNPGN patients without SDH mutations were analysed. Both MCT1 and MCT4 were noted in all the six HNPGN tissue (**Figures 4**, **5**). MCT1 and MCT4 was found to be present in both tumour and stromal regions. No colocalization was observed between MCT1 and MCT4 indicating that the two transporters are expressed in different cell subsets in HNPGN. Further co-localization analysis was performed with concomitant staining with CD68 (macrophage marker) and CD90 (fibroblast marker). Good colocalization of MCT4 with CD68 was observed in all six tissue samples stained indicating that macrophages expressed MCT4 in HNPGN tissue *in situ* (**Figure 5**). MCT1 colocalization with macrophages was observed in two of the three patients with SDH mutations (**Figure 4B**, right panels and data not shown) while none of the samples from patients without SDH mutations showed MCT1 expression in macrophages. In keeping with the low expression of MCT1 and MCT4 on the cultured fibroblasts, limited co-localisation was observed between MCT1/MCT4 and CD90 *in-situ*.

## Discussion

This study reports on macrophage and fibroblast profile in the stromal microenvironment in HNPGN. Here we demonstrate several fibroblast related molecules such as podoplanin and CD90 in HNPGN tissue. We also successfully cultured CD90 expressing fibroblasts from HNPGN. In addition, we demonstrate the presence of M2 macrophages and show expression of metabolic markers MCT1 and MCT4 within macrophages and in tumour cells in HNPGN. To the best of our knowledge this is the first report examining the expression profile of functional and metabolic markers on fibroblasts and macrophages in HNPGN.

CD90, a molecule expressed in fibroblasts, was found both in HNPGN tissue and in the fibroblasts cultured from HNPGN. CD90 or Thy 1 is a small membrane glycophosphatidylinositol (GPI) anchored protein (13) that is involved in cell-cell and cell-matrix interactions, with specific roles in fibroblast proliferation and migration in wound healing, inflammation and fibrosis (14). It is also important to highlight that fibroblasts are a heterogenous cell type and that there is no marker exclusive to fibroblasts. Other than fibroblasts, CD90 is expressed in several cell types including T cells, neurons and pericytes (15, 16). Our immunohistochemistry results as well as the flowcytometry expression of CD90 on cultured HNPGN fibroblasts suggest that CD90 is a good marker to identify fibroblasts in HNPGN; however, its precise functional role in fibroblasts in HNPGN remains to be elucidated. In addition, we found expression of podoplanin (PDPN), another putative fibroblast marker in HNPGN tissue on immunohistochemistry. PDPN is a mucin-type transmembrane glycoprotein of 38-kDa molecular weight and can be demonstrated in a variety of normal cells, e.g. peritoneal mesothelial cells, follicular dendritic cells in lymphoid tissue (17) and is known to be expressed in various tumours (e.g. germ cell tumours and squamous cell carcinoma) (18). Of note PDPN positive fibroblasts are associated with poorer prognosis in some tumours such as lung cancers (19). It is interesting that on immunohistochemistry we found CD90 and PDPN were both expressed in non-lymphatic areas but did not co-localise in HNPGN microenvironment. This suggests that CD90 and PDPN are expressed on different cellular subsets, perhaps different fibroblast subsets in HNPGN. A report in literature suggests that PDPN may be expressed on macrophages and could promote tumour invasion (20). While CD90 expression was noted in cultured fibroblasts, PDPN expression was low in cultured fibroblasts on flowcytometry, which could potentially mean that cultured fibroblasts lose PDPN expression. Fibroblasts derived from HNPGN were also found to express FAP. While cancer associated fibroblasts express many markers, 90% of epithelial tumours show increased expression of FAP in the stroma (21) and this is associated with increased local tumour invasion, increased risk of lymph node metastasis and decreased survival (22). PDPN and FAP expression may have relevance in HNPGN behaviour and will be explored in further large-scale studies. We also found expression of insulin receptor (IR) and insulin growth factor 2-receptor (IGF2-R) on fibroblasts cultured from HNPGN. IR is known to be overexpressed in breast cancer (23), while IGF2-R is correlated to poor prognosis in patients with triple negative breast cancer (24) but their roles in HNPGN is unknown. Of note, overexpression of the related insulin-like growth factor 1 receptor (IGF-1R) has been associated with malignancy in familial pheochromocytomas and paragangliomas (25). However, the specific roles of insulin and insulin growth factors receptors in fibroblasts in HNPGN have not been studied so far.

The presence of CD163 and CD68 positive macrophages in pheochromocytoma and extra adrenal abdominal paragangliomas has been reported previously (26). A recent study has examined immune cell infiltrate in pheochromocytoma and peripheral paragangliomas and reported the presence of macrophages, with the density of the macrophage infiltrate being linked to the aggressive nature of the tumour (27). The latter study however had a small number of HNPGN (2 out of 65 tumours studied) and further subsite delineation of the HNPGN was not described. We show the presence of consistent macrophage infiltration on immunohistochemistry in a larger cohort of HNPGN, and these macrophages displayed a predominantly M2 phenotype as evidenced by CD163 expression. In our cohort of 10 patients, we did not see any correlation between CD163 or CD90 expression and tumour stage, gender or SDH mutational status, however this will need further examination in a larger cohort of patients. In addition, the functional status of the macrophages and fibroblasts may also influence the behaviour of HNPGN and will need future work. A recent paper has examined 32 pheochromocytoma/paraganglioma (PC/PG, including 5 HNPGN) using single cell RNA sequencing and reports that stromal and immune infiltrate can contribute to 0.5 to 76.7% of the cellular composition. Of these, macrophages formed the largest constituent of the immune infiltrate and macrophage markers were found to be expressed at a higher level in PC/PG with SDH and von-Hippel Lindau (VHL) mutations (28).

Our findings and that of the studies above are of great interest as tumour associated macrophages are linked to poor prognosis in several cancers and are actively being explored as targets of cancer immunotherapy (29). Metabolic changes occur in the cancer associated macrophages as well as cancer associated fibroblasts (30, 31). We have examined the functional and metabolic profile of the macrophages and fibroblasts in HNPGN and show that MCT1 and MCT4 are expressed in both the tumour and stromal components of HNPGN. Macrophages in particular express MCT4 in HNPGN, whilst MCT1 expression was also observed in macrophages in HNPGN carrying SDH mutations. Lactate is a major source of carbon for the tricarboxylic acid (TCA) cycle in healthy and cancerous tissue (32). MCT1 is ubiquitously expressed and regulates lactate-H^+^ import. MCT4 is strongly expressed by hypoxic and glycolytic tissue and is mainly responsible for lactate export (33). Several studies indicate that both these molecules are amenable to drug induced alterations and therefore could represent new therapeutic targets in cancer and inflammation (12, 34). The clinical significance of MCT expression has been investigated in several tumours and high MCT1 and MCT4 expression is associated with poor prognosis (34). Macrophages express MCT1 through which they can uptake lactate, in an autocrine or paracrine way, which in turn promotes their differentiation into a regulatory anti-inflammatory phenotype (M2 phenotype). MCT4 expression in macrophages is required for glycolytic reprogramming and inflammatory responses (35). While MCT1 expression was noted on a small proportion of cultured HNPGN fibroblasts, neither MCT1 nor MCT4 co-localised with CD90 *in situ* suggesting that in HNPGN, MCT1/MCT4 driven metabolic changes may be more pronounced in macrophages than in fibroblasts. Our findings that MCT1 and MCT4 are expressed in HNPGN stroma *in situ* suggests a potential role of stromal metabolism in the pathogenesis of these tumours. We highlight however that while our study has described important features of macrophages and fibroblasts in HNPGN with their functional attributes, detailed analysis of the functional relevance of these findings will need to be addressed in future larger cohort studies to establish how these cells influence tumour pathogenesis.

In summary, our results show that fibroblasts and macrophages are an important component of HNPGN tumour microenvironment and express several markers involved in metabolic and cellular interactions. This could shape the tumour stroma interaction in HNPGN and thereby impact on tumour growth. Further dissection of the function of macrophages and fibroblasts in HNPGN could reveal novel strategies to improve patient risk stratification and management by targeting its stromal microenvironment.

## Data Availability

The original contributions presented in the study are included in the article/**Supplementary Material**. Further inquiries can be directed to the corresponding author.

## Supplementary material

Supplementary Figure 1 Expression of CD163 in head and neck paraganglioma (HNPGN) tissue on immunohistochemistry. Immunohistochemistry images of HNPGN tissue (representative of n = 10 HNPGN patient samples) showing CD163 expression in red. The images are labelled PGN02, PGN03 etc denoting origin from different patients. Panels are at 20x magnification.

Supplementary Figure 2 Expression of CD90 in head and neck paraganglioma (HNPGN) tissue on immunohistochemistry. Immunohistochemistry images of HNPGN tissue (representative of n = 10 HNPGN patient samples) showing CD90 expression in brown. The images are labelled PGN02, PGN03 etc denoting origin from different patients.

Supplementary Figure 3 Expression of PDPN in head and neck paraganglioma (HNPGN) tissue on immunohistochemistry. Immunohistochemistry images of HNPGN tissue (representative of n = 10 HNPGN patient samples) showing PDPN expression in red. The images are labelled PGN02, PGN03 etc denoting origin from different patients.

Supplementary Figure 4 Comparison of CD163, CD90 and PDPN quantitative expression. Graphs depict CD90, PDPN and CD163 quantitative expression (each data point represents positive nuclei/area of tissue for individual samples) in the HNPGN samples based on mutation status (upper panels) and gender (lower panels). Supplementary **Figure 4** Analysis of fibroblast and macrophage associated molecules in head and neck paraganglioma (HNPGN). **(A)** Expression of a panel of fibroblasts genes on RT-qPCR in fresh frozen tissue from six human HNPGN samples. Each data point is one patient sample and shows the relative amounts for each gene using GAPDH as the housekeeping gene. **(B)** Expression of a panel of fibroblasts genes on RT-qPCR in fresh frozen tissue from six human HNPGN samples. Each data point is one patient sample and shows the relative amounts for each gene using GAPDH as the housekeeping gene.

Supplementary Figure 5 Fibroblasts derived from head and neck paraganglioma (HNPGN) and normal skin/mucosa. **(A)** Phase contrast photomicrography images of fibroblasts cultured from fresh HNPGN tissue (representative of n=10 HNPGN patient samples) and normal mastoid mucosa/ear canal skin tissue (representative of n=6 patient samples). **(B)** RT-qPCR was used to quantify the expression of macrophage associated molecules on the HNPGN and normal fibroblasts (representative of n=3-4 paired samples). Panels show expression of genes of the galectin (LGALS1, LGALS9, LGALS3, LGALS3BP) family, CTSB, CTSL1, matrix metalloproteinases (MMP2, MMP14), CxCL16, MYC, EPAS1, SUMO1, TNS3, PIAS, and HTR2. Each data point is one sample and shows the relative amounts for each gene using GAPDH as the housekeeping gene.
