# Supplementary material for: Functional analysis of fibroblasts and macrophages in head and neck paragangliomas
Source: Front Endocrinol (Lausanne). 2024 Nov 15;15:1397839. doi: 10.3389/fendo.2024.1397839 (PMC11604431; doi:10.3389/fendo.2024.1397839)

Supplementary Table 1: Patient demographics

Patient number	Age	Sex	Tumour type	Mutation	Stage	Outcome
PGN01	34	M	Glomus vagale	None	Group A (NG)	Malignant/distant metastases
PGN02	53	F	Glomus tympanicum	None	Group B (F)	No recurrence
PGN03	37	F	Glomus jugulare	None	Group C1 (F)	Stable residuum
PGN04	26	M	Glomus jugulare	SDHB	Group D (F)	Stable residuum/vagal palsy
PGN05	39	F	Glomus jugulare	None	Group C1/2 (F)	Stable residuum
PGN06	51	F	Glomus tympanicum	None	Group B (F)	No recurrence
PGN07	50	F	Glomus jugulare	None	Group B (F)	Growing residuum
PGN08	72	F	Glomus jugulare	None	Group B (F)	Stable residiuum/facial palsy
PGN09	28	F	Glomus jugulare	SDHB	Group C1 (F)	Stable residuum/vagal palsy
PGN10	42	M	Glomus jugulare	SDHD	Group B (F)	No recurrence/Facial palsy

NG: Netteville Glasscock Classification

F: Fisch Classification

Supplementary Figure 1

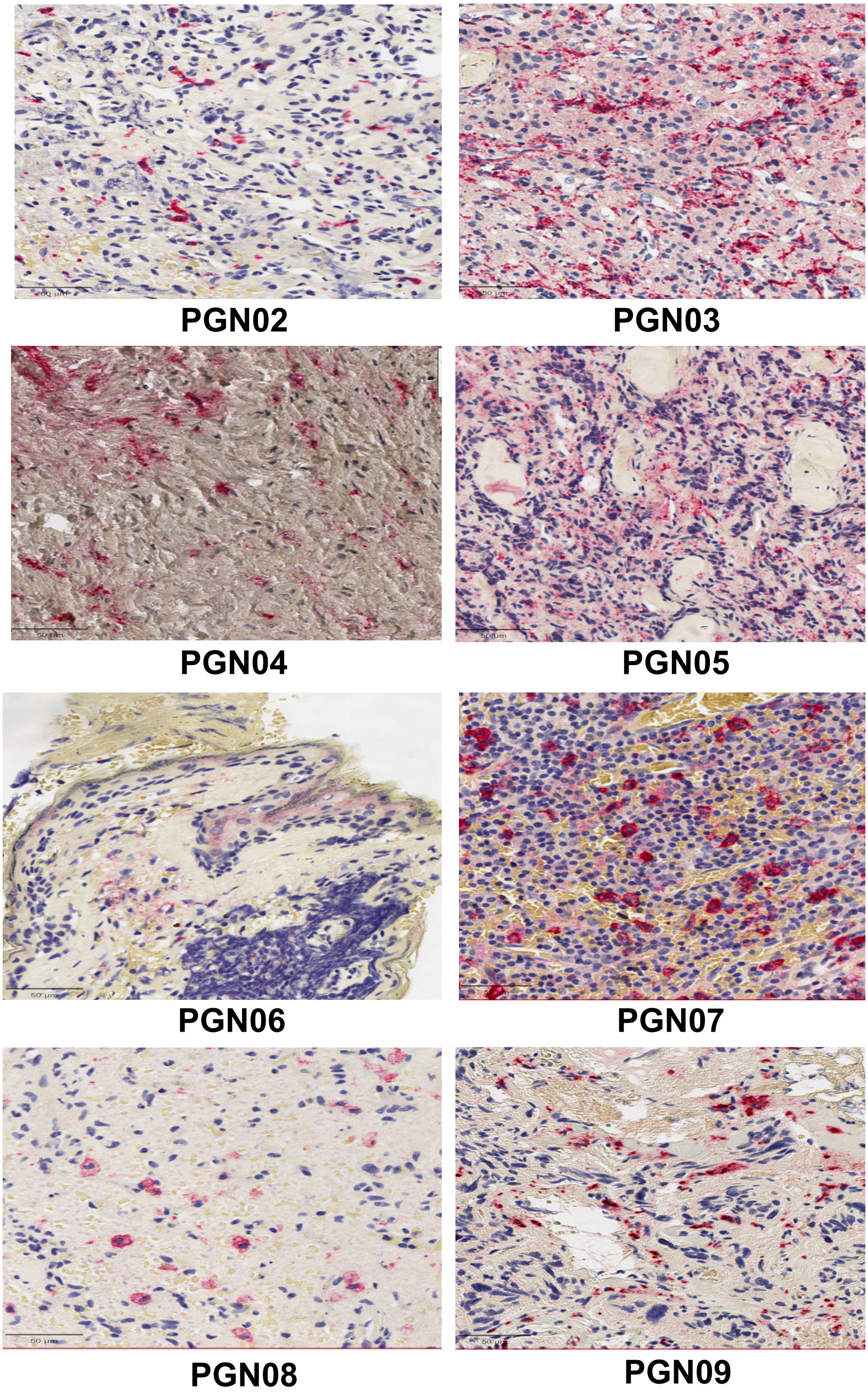

Supplementary Figure 2

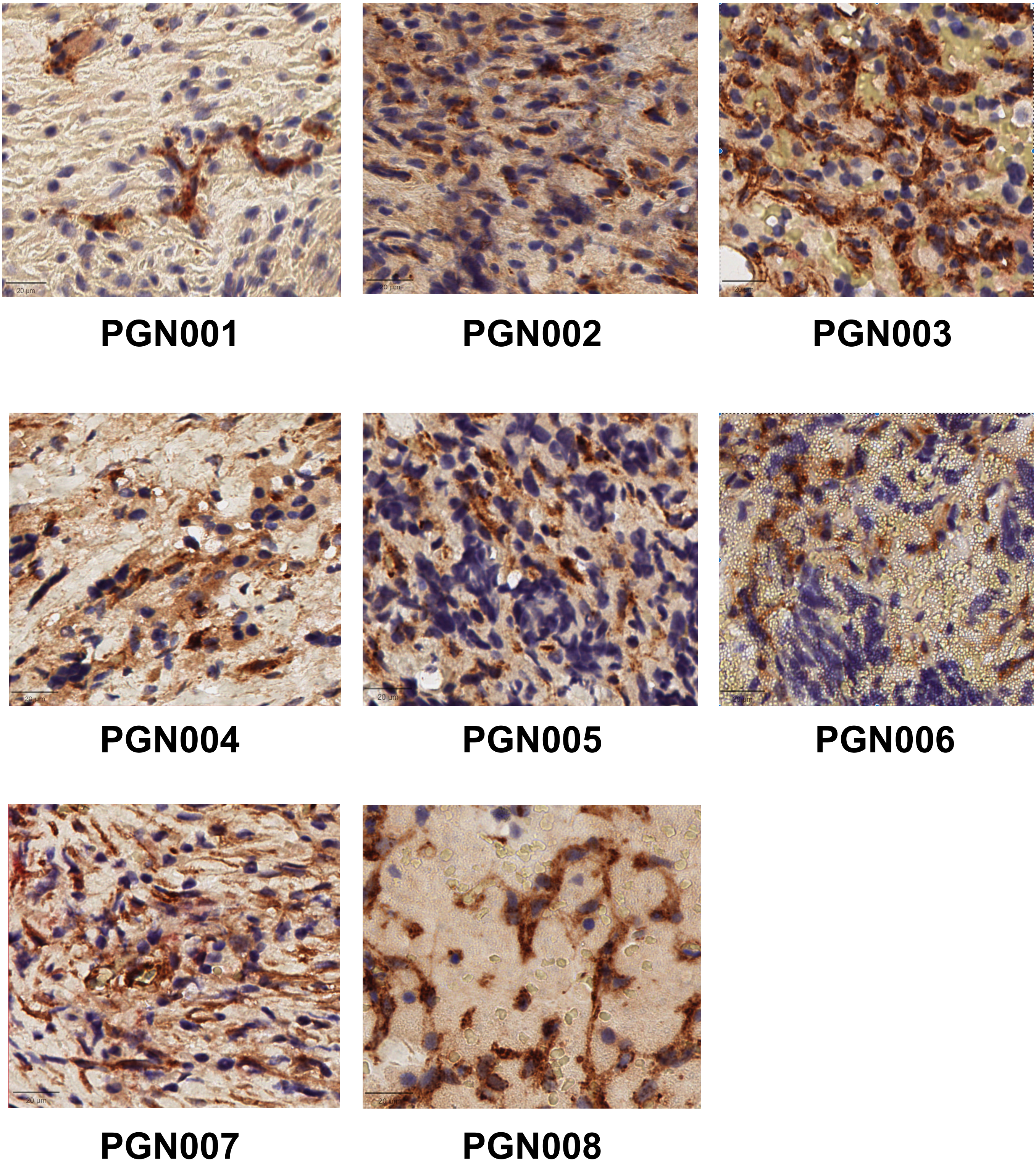

Supplementary Figure 3

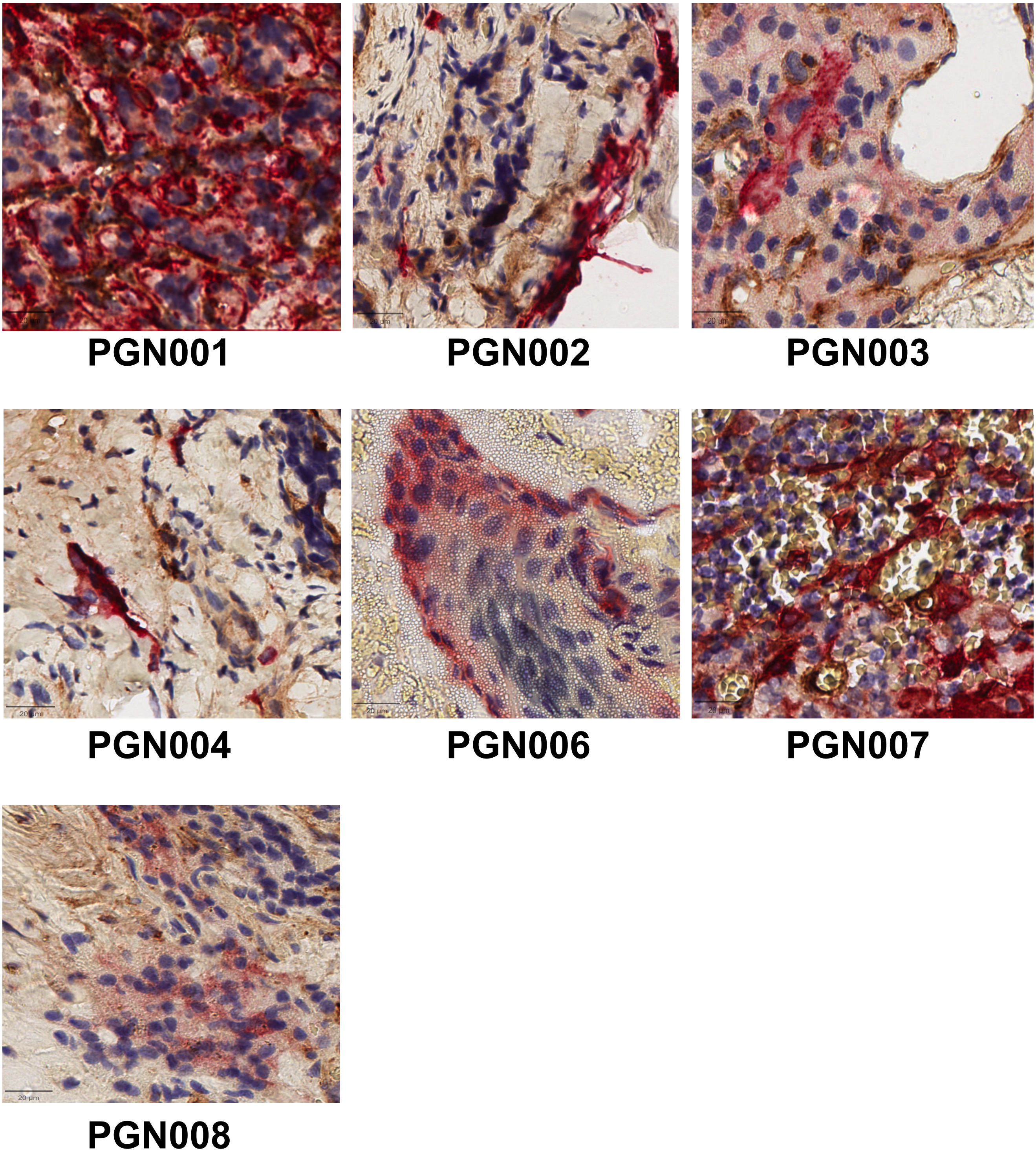

Supplementary Figure 4

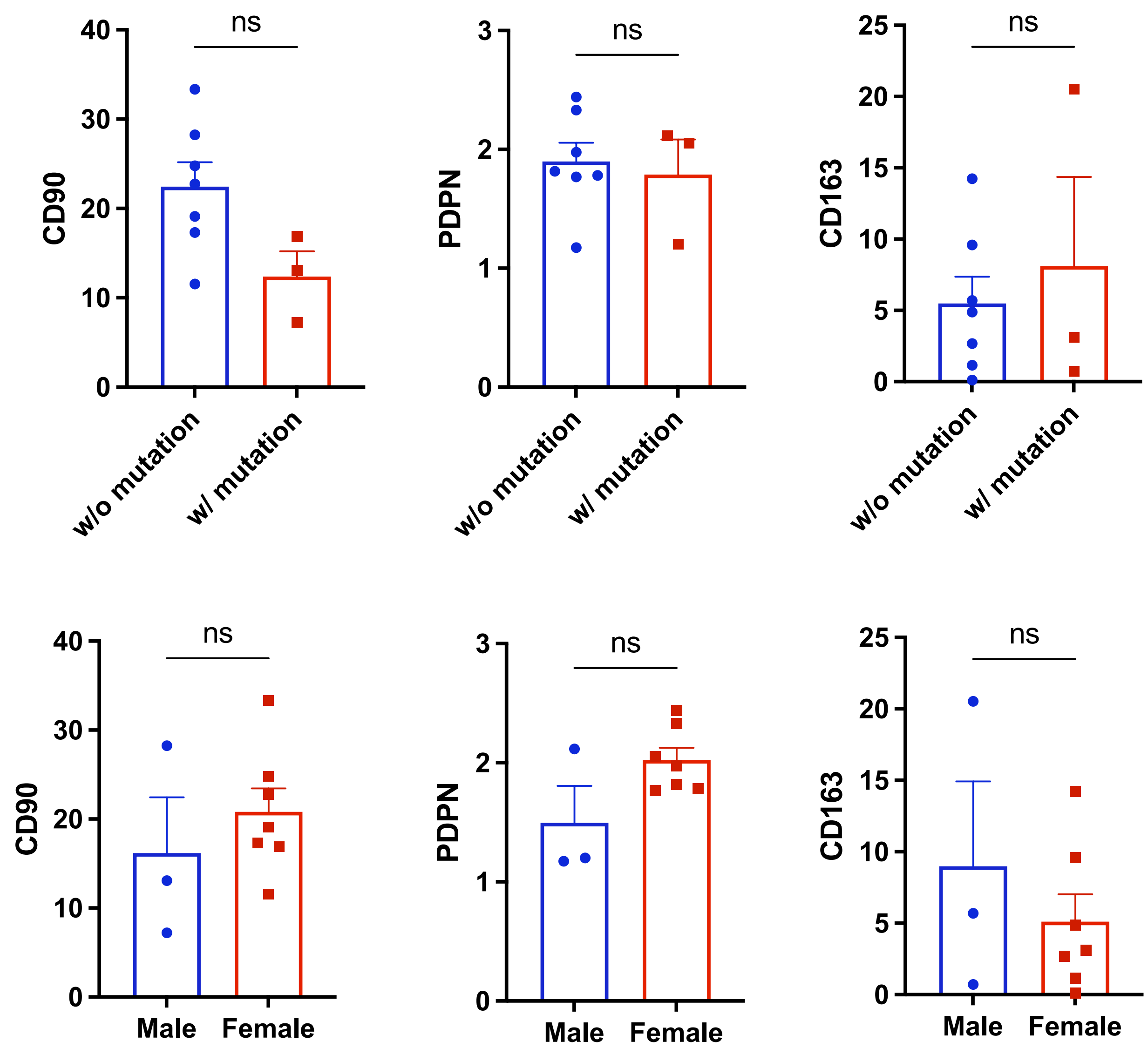

Supplementary Figure 5

A

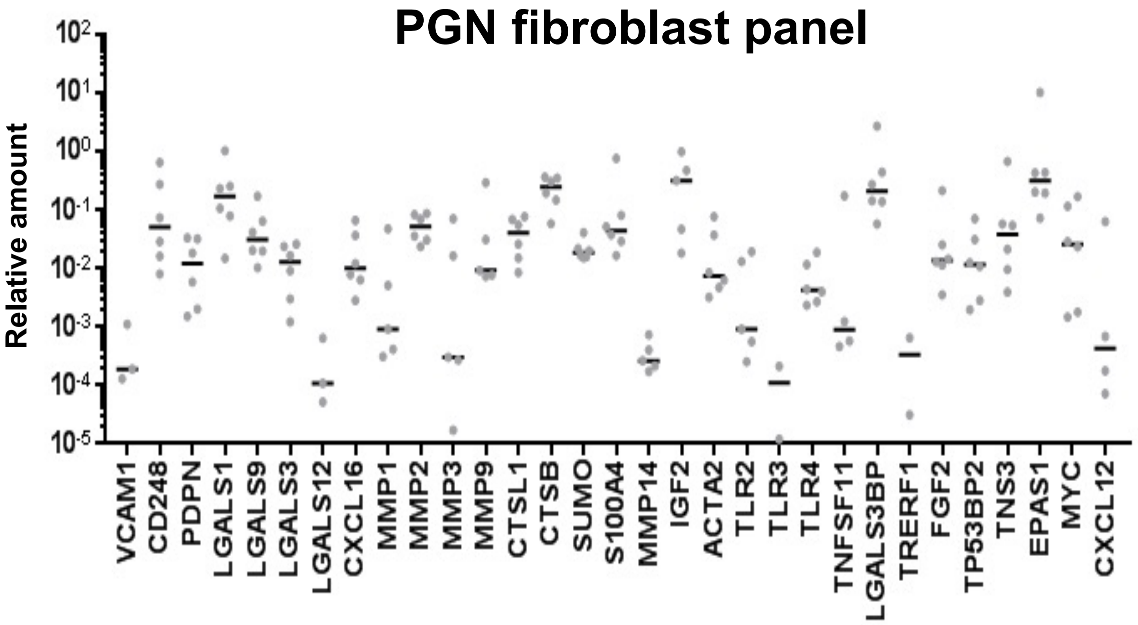

B

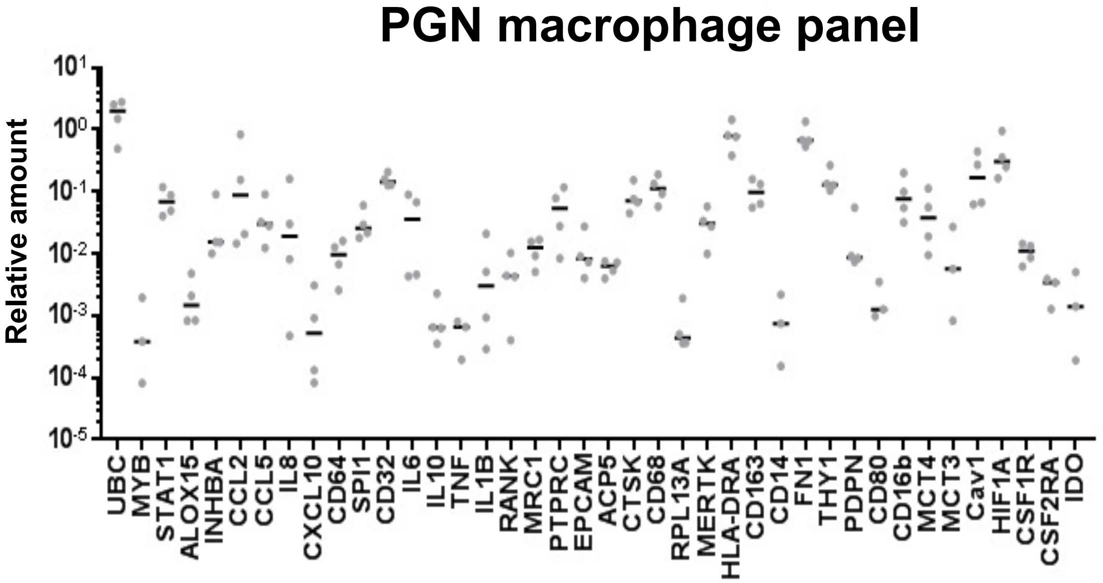

Supplementary Figure 6

A

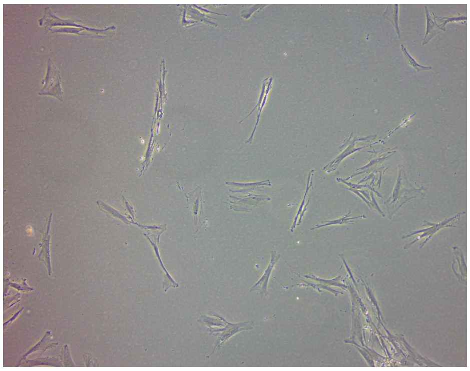

PGN fibroblasts

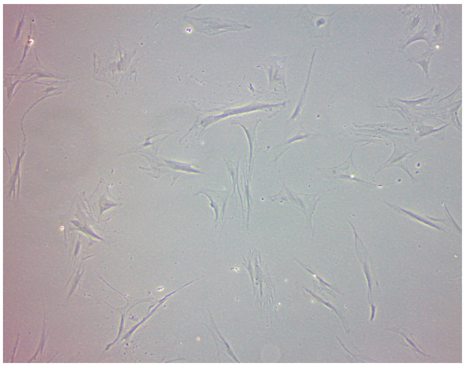

Mucosal fibroblasts

B

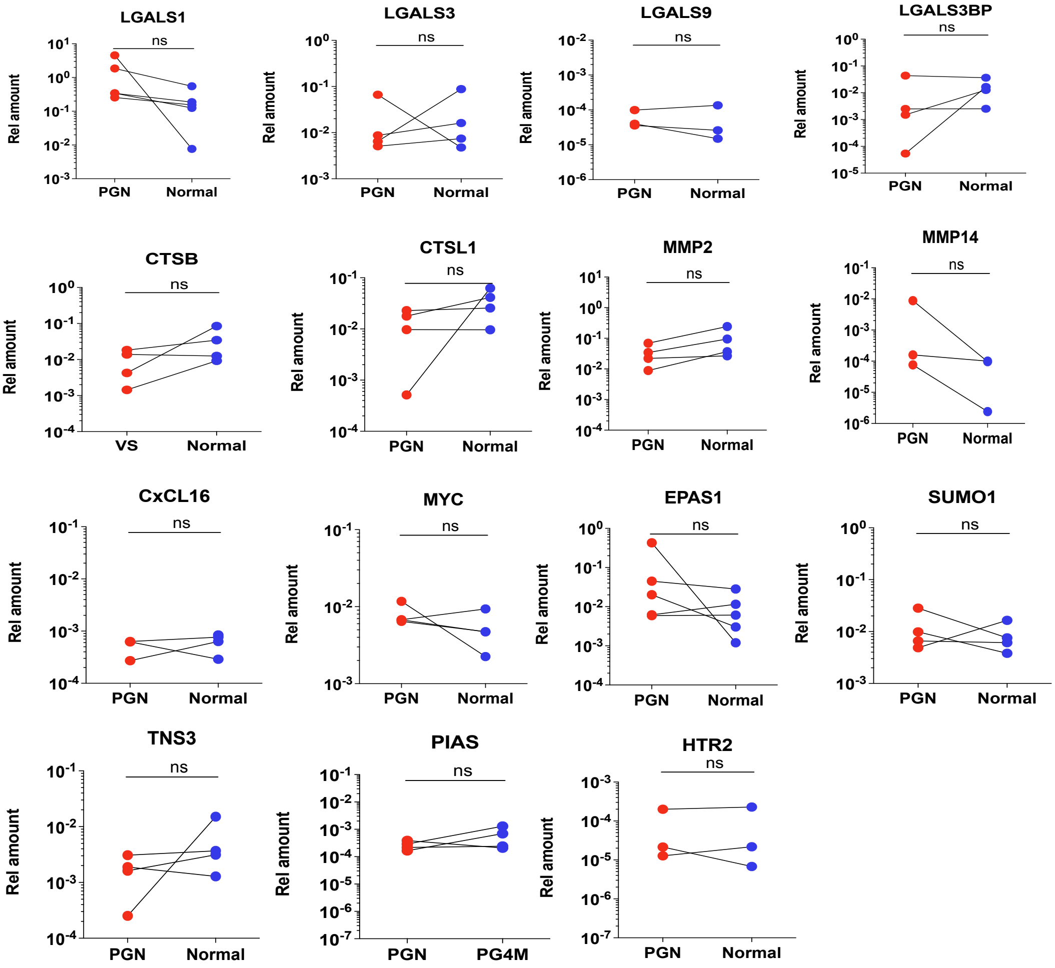

Supplement: Supplementary Figure 1 — Expression of CD163 in head and neck paraganglioma (HNPGN) tissue on immunohistochemistry. Immunohistochemistry images of HNPGN tissue (representative of n = 10 HNPGN patient samples) showing CD163 expression in red. The images are labelled PGN02, PGN03 etc denoting origin from different patients. Panels are at 20x magnification. [file DataSheet1.pdf]
